# Leakage-Controlled and Survey-Weighted Machine Learning for Neonatal Mortality Risk Prediction Using NFHS-5 Data

**DOI:** 10.3390/healthcare14142144

**Published:** 2026-07-16

**Authors:** Moumita Mukherjee, Talha Ali Khan, Raja Hashim Ali

**Affiliations:** 1 Institute of International Health, Charité—Universitätsmedizin, 13353 Berlin, Germany; 2 Department of Business, University of Europe for Applied Sciences, 14469 Potsdam, Germany; talhaali.khan@ue-germany.de (T.A.K.); hashim.ali@ue-germany.de (R.H.A.)

**Keywords:** neonatal mortality, NFHS-5, machine learning, survey weighting, rare-event prediction, calibration, geographic validation

## Abstract

**Background:** Neonatal mortality remains uneven across Indian states, while prediction studies using survey data are often limited by class imbalance, data leakage, inadequate calibration, and insufficient consideration of complex survey design. This study developed and rigorously evaluated survey-aware machine-learning models for neonatal mortality risk prediction using NFHS-5 data. **Methods:** Data from 33,338 children in Bihar, Chhattisgarh, and Uttarakhand were analysed. Household-grouped development/test splitting, repeated grouped nested cross-validation, DHS sampling weights, fold-contained preprocessing, socioeconomic clustering, particle swarm optimisation, resampling, and out-of-fold feature augmentation were applied. Logistic regression, random forest, histogram gradient boosting (HGB), and artificial neural networks were compared using PR-AUC as the primary metric. Calibration, household-bootstrap confidence intervals, decision-curve analysis, prediction timepoint analysis, and leave-one-state-out validation were performed. **Results:** HGB achieved the highest repeated cross-validation PR-AUC (0.189) and ROC-AUC (0.778). On the untouched test set, ROC-AUC was 0.755 (95% CI 0.718–0.796), and PR-AUC was 0.202 (0.138–0.267), with sensitivity 0.813, specificity 0.517, PPV 0.063, and NPV 0.989. Clustering, PSO, SMOTE, and augmentation added little value. Antenatal performance was weaker, and state-wise transportability varied. **Conclusions:** Survey-weighted HGB provided the strongest predictive performance, but low PPV and heterogeneous state-level results restrict its use to low-cost screening. Prospective validation is required before deployment.

## 1. Background

The overall rate of neonatal mortality in India is 17 per 1000 live births in 2024 as per the World Bank data, and more than one-fourth of neonates (26%) died in the first 24 hours of birth considering the neonatal deaths after live birth [1,2]. The neonatal mortality varies by province as per the report of National Family Health Survey 2019–2021 [3]. States such as Bihar, Uttarakhand, Chhattisgarh, and Uttar Pradesh depict very high rates (above the rate of 30 per 1000 live births) [3]. Furthermore, the inequity in access to maternal and child health services is prominent in states that are lagging in achieving health outcomes [3]. Studies reflect less efficient resource allocation; traditional methods of generating insights from available data are the correlates of reduced programme effectiveness in achieving outcomes [4].

### 1.1. Evidence from Classical Analytics

The current study explores both the classical and innovative schools of thought. From diverse classical empirical studies exploring the linkage between neonatal survival outcomes and social determinants found factors like previous history of failed pregnancy/childbirth, factors related to maternal healthcare access and lifestyle factors as major determinants. A mixed-effect multivariate analysis applying four consecutive Ethiopian DHS datasets explored the trend of infant mortality and its determinants [5]. The study by [6] tested the association between survival status of preceding children and risk of mortality in successive ones along with other determinants, using the Cox Proportional Hazard model. A systematic review and meta-analysis exploring the timing of neonatal mortality and severe neonatal morbidity covered 36 reports consisting of observational studies (cross-sectional, cohort, prevalence, prospective) in 51 study settings, including a total of 6,760,731 live births and 47,551 neonatal deaths [7]. Another systematic review and meta-analysis evaluating the association between birth spacing, and the adverse pregnancy outcomes on a global scale, reviewed 129 observational studies (cross-sectional, cohort, case-control) and experimental (cohort) studies comprising 46,874,843 participants [8]. Another study investigated the relation between seven ‘quality of diet’ scores in pregnancy, birth and child health outcomes in the form of a narrative review [9]. A systematic review and meta-analysis of 81 maternal and birth cohort studies in Gulf countries (Bahrain, Kuwait, Oman, Qatar, Saudi Arabia, and the United Arab Emirates) analysed the association between maternal obesity and large-for-gestational-age newborns [10]. A cross-sectional study by [11] explored the association between ethnicity, teenage pregnancy, and adverse birth outcomes in Suriname using national level and a few facility level data. The study by [12] explored the determinants of adverse birth outcomes in Sub-Saharan Africa using data from 76,853 children. Another research study conducted a scoping review of 10 studies that explored the preconception risk factors and interventions related to teenage pregnancy to reduce adverse pregnancy and birth outcomes [13]. Different facility-based cross-sectional studies or community-based studies in different low-and middle-income countries worth mentioning here explored the correlates of adverse birth outcomes—stillbirth, preterm birth, low birth weight occurring after premature rupture of membrane—as PROM mothers are more likely to have adverse birth outcomes, which is evident in 3% to 8% of pregnancies globally, contributing to neonatal mortality [14,15,16,17]. Another study by [18] investigated the association between exposure to malaria and the risk of adverse maternal health, pregnancy and birth outcomes from 253 studies.

In addition to this further, the evidence from a number of recent studies depict higher effectiveness of machine learning and deep learning techniques over classical modelling techniques in exploring the determinants of health outcomes, with manual model adjustments for the classification of outcomes [19,20,21,22,23,24,25].

### 1.2. Evidence from the Application of Advanced Analytics—Machine Learning and Deep Learning

The studies grouped under the theme of innovative and translational science benefit approach tested novel methods to improve public health service delivery through enhancing the quality of data gathering, storage, analysis, and decision-making [26]. Such literary works reflect that improvement in healthcare decision support focuses on quality improvement in clinical decision-support systems (CDSS) and has reached developing stage of digital health maturity from the initial ideation level in most of the applications. Some of the studies build a use case to optimise public health decision support and propose models to adopt the concept for faster and more accurate analysis of routine data using machine learning and deep learning. The process trains a system, then is tested to find the best model as per the evaluation metrics [22,23,24].

These models are mainly used to access past decisions, identify contextual variables, predict and suggest better solutions to learning, and make decisions to improve implementation [22,26,27]. In healthcare, CDSSs are mainly used for knowledge and information management applying generally rule-based logic [28,29,30] or algorithms [29,31], and some use neural networks [32,33,34]. Evidently, the most used algorithms are artificial neural networks (ANN), support vector machines (SVM), and decision trees (DTs) in proposing CDSSs in different settings [35]. In low-and middle-income countries, DSSs are used to strengthen public health services in the areas of health surveillance, evaluation of programmes, and predictive modelling [36]. Previous studies indicated that public health DSSs (PHDSSs) require an approach to ensure their effectiveness. In some of the better-performing states in India, PHDSSs have already shown their effectiveness in improving access to care through pilot interventions [26,37,38].

India has started deploying state-of-the- art technology to create and test the feasibility of DSS in the public health sector to ensure equitable access to basic health services, including MCH services. Among the different initiatives, SMARThealth Pregnancy—a CDSS using a mobile application—was aimed at reducing the cardiovascular risks associated with pregnancy and implemented in rural areas of India covering primary health centres in Jhajjar district, Haryana, and Guntur district in Andhra Pradesh, as a pilot and feasibility study [26]. The integrated mobile CDSS applied simple algorithmic rules, collecting data at point of care on anaemia, haemoglobin, oral glucose tolerance test, and heart rate, along with historical data on the pregnant women, to generate signals based on traffic colour coding. This mobile CDSS model was found to be a successful decision-support system applied from the comfort of the homes of the pregnant women and used by community health workers in their routine activities. It helped to identify high-risk pregnancies and tailor ANC requirements for them—depicting higher efficiency and effectiveness—and improving the quality of service [26]. Reproducible algorithms are applied to explore the feasibility of smart analytic-driven PHDSS or CDSS in identifying factors behind high rate of caesarean deliveries or in detecting anomalies with higher accuracy [39]. The study by [40] proposed a DSS to identify COVID-19 risk factors with higher accuracy and precision. A study by [41] demonstrated the effectiveness of a smart mobile application-based CDSS for community health workers in managing non-communicable diseases in rural India. In a related study, a case-control pilot evaluated an mHealth application-based PHDSS across two primary health centres in northern India [42]. The intervention group was exposed to a decision tree algorithm-based mobile application as point-of-care use by community health workers to record patient demographics, history of morbidity, and examination and investigation details, to generate a birth plan after identifying the risks. The study shows significant differences in triage assessment, ANC uptake and counselling among the case and control groups, where the control group participants received a conventional service. This provides evidence of the higher effectiveness of PHDSSs in rural areas of India.

### 1.3. Application of ML and DL in Improving MCH Services

A systematic review explored the usability of ML and/or DL models in improving the predictability of neonatal, infant, perinatal mortality or stillbirth outcomes from earlier research, and found that 83% of the selected research used ML algorithms and 17% used DL [43]. Different studies focused on neonatal mortality used ML or DL for classifying the occurrence of neonatal death and mostly considered birth weight, gestational age, child’s sex, mother’s age, educational level, ethnicity, heart rate, number of ANC visits, history of previous pregnancies (type of pregnancy, type of delivery, number of healthy and stillbirths), and birth order as predictors [43,44,45,46,47,48,49,50,51]. Almost 60% of the studies focused on classifying the occurrence of neonatal outcomes used random forest, 50% used neural networks, 50% used SVM, 25% used k-NN, 17% used XGBoost or GBM, and less than 10% used ensemble methods [43].

### 1.4. Feature-Augmented Models and Benefits over Basic Models

Different studies under classical analytics have shown how factors such as short birth interval increase the risk of repeated preterm birth with nutritional shortage, Again, the risk of preterm birth after pregnancy loss with a short birth interval is more likely and the higher occurrence of preterm birth increases the risk of more adolescent pregnancies with poor obstetric history, placing teenage married girls in a locus of early marriage, early pregnancy, preterm birth, failed pregnancy, and recurrent preterm births leading to adverse pregnancy and/or birth outcomes [52,53]. These studies raise concerns in the classical school regarding the need for mediation analysis. Different studies have shown that augmented models outperform single or ensembled basic models in advanced analytics. For example, [54] inferred that an augmented model brought 99.67% accuracy compared to other models, such as SVM and RF, with less time in processing. A study using a deep convolutional neural network (CNN) with the incorporation of transfer learning and data augmentation improved the classification of progressive neurodegenerative disease [55]. In this study, the authors gathered 504 images, and 360 images were used for data augmentation; they ultimately worked on 4200 images and built a model with 89% classification accuracy. In another study, the use of a sparse encoder with CNN improved the classification accuracy by 3% from conventional algorithms, where they achieved 91.3% accuracy in predicting diabetes among the population [56]. Another study classified the diabetic population by applying four augmentation techniques with three neural network algorithms and achieved more than 95% accuracy compared to other models that used only blood glucose level as model input [57].

### 1.5. Gap Analysis

Despite growing use of machine-learning and deep-learning methods for neonatal mortality prediction, important methodological gaps remain. Preprocessing, feature selection, clustering, and resampling are not always restricted to training data, creating a risk of data leakage and optimistic performance estimates. Evaluation also often relies too heavily on accuracy and ROC-AUC, although neonatal mortality is a rare outcome that requires PR-AUC, sensitivity, specificity, predictive values, calibration, and uncertainty estimates. In addition, conventional random splitting may place children from the same mother or household across development and test sets and ignore DHS sampling weights and clustered survey structure. Finally, complex combinations of clustering, PSO, SMOTE, feature augmentation, and neural networks are frequently presented without fair comparison against simpler baselines or the controlled ablation of each component. These limitations motivate a household-grouped, leakage-controlled, survey-aware evaluation framework that compares simple and complex models under identical validation conditions.

### 1.6. Research Questions

The research questions addressed under this study are as follows:

**RQ1.**  *How accurately can neonatal mortality be predicted using maternal, socioeconomic, healthcare access, pregnancy, and birth-related NFHS-5 variables under a household-grouped, leakage-controlled validation protocol?*

**RQ2.**  *How do logistic regression, random forest, histogram gradient boosting, and artificial neural networks compare under an identical repeated grouped-validation framework?*

**RQ3.**  *What are the individual contributions of socioeconomic clustering, PSO feature selection, SMOTE, and out-of-fold feature augmentation?*

**RQ4.**  *How does predictive performance change when models are restricted to antenatal, delivery time, or retrospective information?*

**RQ5.**  *How well does the selected model transport across the three study states?*

**RQ6.**  *Which prespecified maternal, pregnancy, socioeconomic, and healthcare access variables are associated with neonatal mortality in a survey-weighted reference analysis?*

### 1.7. Novelty of This Study

The novelty of the present study lies not in introducing a new algorithm, but in developing a rigorous, survey-aware, and leakage-controlled modelling framework for neonatal mortality prediction using NFHS-5 data. The principal methodological contributions are as follows:

- Applying household-grouped development and test partitions to prevent records from the same household or mother from appearing in both datasets;
- Using repeated grouped nested cross-validation to ensure that model selection and performance estimation are conducted under a consistent validation protocol;
- Fitting all preprocessing, socioeconomic clustering, particle swarm optimisation, feature augmentation, calibration, and resampling procedures exclusively within the corresponding training folds;
- Incorporating DHS sampling weights during the training and evaluation of eligible models and accounting for the clustered survey structure in a separate association analysis;
- Comparing logistic regression, random forest, histogram gradient boosting, and artificial neural networks under the same validation framework;
- Conducting a controlled ablation analysis to quantify the individual contributions of socioeconomic clustering, PSO feature selection, SMOTE, and out-of-fold feature augmentation;
- Prioritising precision–recall area under the curve as the primary selection metric and reporting sensitivity, specificity, positive and negative predictive values, F1-score, calibration, and decision-curve net benefit;
- Generating household-grouped bootstrap confidence intervals to quantify uncertainty in final-test performance;
- Distinguishing antenatal, delivery time, and retrospective prediction settings according to the real-world availability of input variables;
- Evaluating geographic transportability through leave-one-state-out validation across Bihar, Chhattisgarh, and Uttarakhand;
- Clearly separating predictive modelling from non-causal survey-weighted association analysis and from the future development of an operational decision-support system.

### 1.8. Significance of Our Work

The significance of this study lies in providing a more credible and practically interpretable assessment of neonatal mortality prediction than would be obtained from a conventional random split or accuracy-centred evaluation. The final results demonstrate that histogram gradient boosting performed better than logistic regression, random forest, and artificial neural networks, whereas the addition of clustering, PSO, SMOTE, and feature augmentation produced limited independent improvement. This finding is important because it shows that increasing modelling complexity does not necessarily improve prediction in structured population-survey data. The study also demonstrates that predictive performance depends strongly on the time at which information becomes available: antenatal prediction remained comparatively weak, while delivery time variables substantially improved discrimination. Although the selected model achieved high sensitivity and negative predictive value, its low positive predictive value indicates that it should be considered only as a potential low-cost screening aid rather than a diagnostic or automatic referral system. The framework therefore provides an evidence base for future prospective validation, state-specific recalibration, and integration with HMIS or community health worker workflows, while avoiding unsupported claims of causal effects, improved neonatal outcomes, or an already implemented decision-support system.

## 2. Methods

### 2.1. Study Design and Analytical Scope

This study conducted a secondary analysis of the Indian DHS National Family Health Survey 2019–2021 (NFHS-5) to develop and evaluate machine-learning models for neonatal mortality risk prediction. The primary analytical objective was predictive rather than causal. Accordingly, the main analysis compared several machine-learning models under a household-grouped, leakage-controlled, and survey-aware validation framework. A separate survey-weighted regression analysis was performed to examine the adjusted associations between prespecified maternal, socioeconomic, pregnancy, healthcare access factors and neonatal mortality. This association analysis was not interpreted as evidence of causal effects. The study did not implement or prospectively evaluate an operational decision-support system; instead, it assessed whether the resulting prediction framework could provide a methodological basis for future low-cost neonatal risk-screening applications.

### 2.2. Data Source, Study Setting, and Study Population

Data were obtained from the Kids Recode file of the NFHS-5, collected in India between 2019 and 2021 [3]. The analysis focused on Bihar, Chhattisgarh, and Uttarakhand because these states reported comparatively high neonatal mortality and represented distinct eastern, central, and northern Indian settings. Eligible records were restricted to children with sufficient information to define neonatal survival status and the required maternal, household, pregnancy, delivery, and healthcare access variables. After applying the study eligibility criteria and data-quality checks, the final analytical sample comprised 33,338 child records.

**Figure 1:**
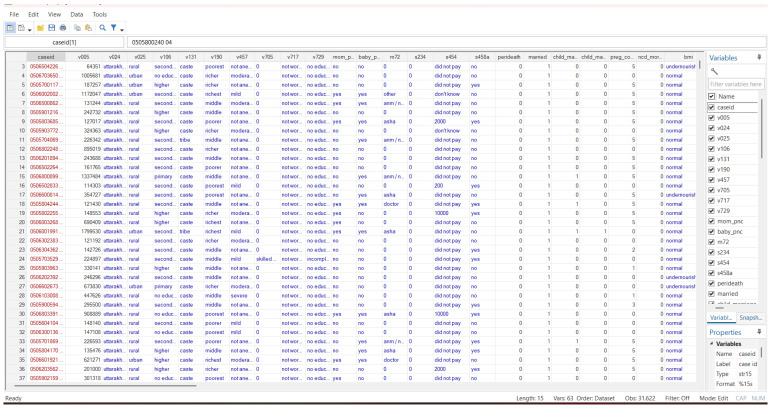
An excerpt of the NFHS-5 analytical dataset, used in the study.

NFHS-5 used a stratified multistage cluster-sampling design. The analysis therefore retained the DHS sampling-weight variable, primary sampling unit identifier, survey strata identifier, household identifier, and mother identifier. These variables were used to support survey-aware estimation, household-grouped validation, and verification that related observations were not divided between the development and final-test datasets. Figure 1 displays an excerpt of the NFHS-5 analytical dataset used in the study.

### 2.3. Outcome Definition

The primary outcome was neonatal mortality, defined as death within the first 28 completed days after birth. The outcome was encoded as a binary variable, where 1 represented neonatal death, and 0 represented survival beyond the neonatal period. Records with missing or internally inconsistent outcome information were excluded before model development. Because neonatal mortality represented a small proportion of the observations, it was treated as a rare-event classification problem throughout model development and evaluation.

### 2.4. Predictor Domains and Variable Construction

Candidate predictors were selected from established determinants of neonatal health and were organised into five domains: maternal and demographic characteristics, socioeconomic and household conditions, maternal health and behavioural characteristics, pregnancy and obstetric history, healthcare access, delivery, and neonatal characteristics. Derived variables included maternal-age categories, birth interval categories, maternal body mass index groups, anaemia status, history of failed pregnancy, antenatal-care uptake, institutional delivery, birth attendance indicators, postnatal care indicators, sanitation, water source, health insurance coverage, and Janani Suraksha Yojana assistance. Variables were retained only when they could be derived consistently from the NFHS-5 data dictionary and had a plausible temporal relationship with the prediction setting under consideration. Variables that directly determined an intermediate target were excluded from the corresponding first-stage prediction model to prevent target leakage.

### 2.5. Data Audit, Distribution Assessment, and Missingness

**Figure 2:**
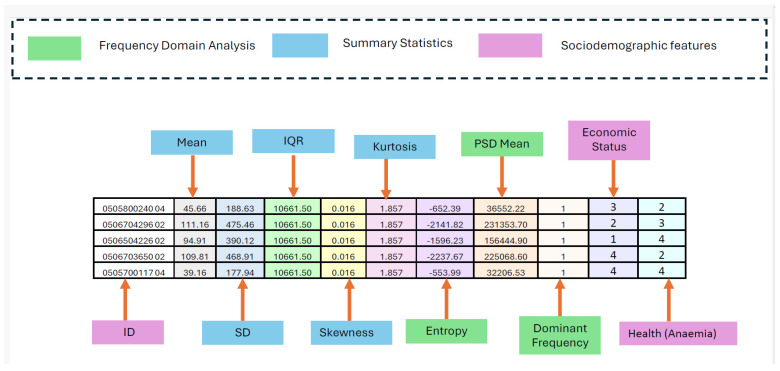
Data audit summary showing the distributions and missingness patterns of selected maternal, pregnancy, socioeconomic, healthcare access, and neonatal variables before model development.

Before model development, the analytical dataset was examined for outcome prevalence, missingness, implausible values, category sparsity, and the distributions of continuous and categorical variables. Numerical predictors were imputed using statistics estimated from the training data, whereas categorical variables were imputed using training-derived category frequencies or most-frequent values. Missingness indicators were retained for selected clinically important variables when the absence of measurement could itself reflect healthcare access or data collection processes. Categorical variables were one-hot encoded, and numerical scaling was applied where required by the corresponding learner. All imputations, encoding, scaling, and missingness transformations were fitted exclusively on the training portion of each validation fold and then applied to the corresponding held-out observations. The data audit summary is illustrated in Figure 2.

### 2.6. Complex Survey Design and Grouping Structure

The NFHS-5 sampling weight was normalised and used during the fitting of eligible non-resampled models and during weighted final-test evaluation. Primary sampling unit and survey strata identifiers were retained for the survey-weighted association analysis. Household and mother identifiers were used to prevent related records from appearing in both development and test partitions. This was necessary because children from the same household or mother may share socioeconomic, behavioural, environmental, and healthcare access characteristics. For the survey-weighted association analysis, uncertainty was estimated using stratified primary sampling unit bootstrap resampling. Regularisation was applied for numerical stability, and the resulting coefficients were interpreted only as adjusted associations.

### 2.7. Household-Grouped Development and Final-Test Split

The analytical sample was divided into development and final-test datasets using household as the grouping unit. All children belonging to the same household were assigned to the same partition. Mother identifiers were subsequently checked to confirm that no mother appeared in both datasets. The outcome distribution was approximately preserved across partitions where feasible. The final-test dataset was isolated before preprocessing, feature selection, clustering, resampling, calibration, threshold selection, or model tuning. It was used only once for final evaluation after the modelling pipeline had been selected using the development data.

### 2.8. Prediction Timepoint Definitions

Three prediction settings were evaluated to distinguish model performance according to the real-world availability of input information. The antenatal model included only maternal, socioeconomic, household, obstetric history, and healthcare access variables that could reasonably be known before delivery. The delivery time model additionally included information available during or immediately after childbirth, such as gestational duration, delivery setting, and birth-related characteristics. The retrospective model included the complete eligible predictor set, including postnatal information, and was treated as an upper-bound benchmark rather than an early-warning model. This separation prevented retrospective variables from being incorrectly presented as inputs for antenatal screening.

### 2.9. Socioeconomic Clustering

Socioeconomic clustering was evaluated as an optional contextual feature-engineering component. State, place of residence, wealth category, and maternal education were used to derive socioeconomic groupings. Nominal variables were one-hot encoded before K-means clustering, and the cluster model was fitted only on the training data within each validation fold. Cluster assignments for held-out observations were generated using the corresponding training-fitted cluster model. Clustering was included in the controlled ablation analysis to determine whether it provided predictive information beyond the original socioeconomic variables.

### 2.10. Particle Swarm Optimisation for Feature Selection

Binary particle swarm optimisation was used to evaluate candidate predictor subsets. Each particle represented a binary feature-inclusion mask. Binary particle swarm optimisation used 12 particles and 12 iterations, with inertia, cognitive, and social coefficients of 0.72, 1.49, and 1.49, respectively. Candidate subsets were evaluated using three-fold cross-validated precision–recall area under the curve within the corresponding training data. A feature-count penalty was included to discourage unnecessarily large subsets. PSO was rerun independently within each training fold. Validation and test observations were never used to select features. Convergence histories and selected feature sets were retained to support reproducibility.

### 2.11. Out-of-Fold Feature Augmentation

Feature augmentation was evaluated using predicted probabilities for four intermediate outcomes: preterm birth, low birth weight, failed pregnancy history, and teenage or late pregnancy. For each intermediate outcome, direct source or proxy variables were removed from the corresponding first-stage model. Household-grouped out-of-fold predictions were generated for the development data, while held-out predictions were obtained from models fitted only on the corresponding training data. These predicted probabilities were then evaluated as additional inputs to the neonatal mortality classifier. They were treated as predictive features rather than mediators, and no causal interpretation was assigned to them.

### 2.12. Class Imbalance Handling

Four training strategies were compared: no resampling, random undersampling, random oversampling, and synthetic minority oversampling. Resampling was performed only after preprocessing the training portion of each fold. Validation and final-test observations were never resampled or used to generate synthetic observations.

Because synthetic observations do not inherit natural DHS sampling weights, resampled models were treated as methodological sensitivity analyses. Survey weights were used during fitting for eligible non-resampled models and during final weighted evaluation.

### 2.13. Candidate Predictive Models

Four model families were evaluated under the same household-grouped validation framework:

1. Logistic regression;
2. Random forest;
3. Histogram gradient boosting;
4. Artificial neural network.

The same development partitions and primary model selection criteria were used for all learners. Model complexity was not assumed to confer superior performance, and the final learner was selected empirically.

### 2.14. Controlled Ablation Analysis

A controlled ablation analysis was conducted to estimate the marginal contribution of each modelling component. The following specifications were evaluated sequentially:

1. Baseline logistic regression;
2. Logistic regression with socioeconomic clustering;
3. Logistic regression with PSO-selected features;
4. Logistic regression with clustering and PSO;
5. Logistic regression with clustering, PSO, and SMOTE;
6. Logistic regression with clustering, PSO, and out-of-fold feature augmentation;
7. The complete logistic regression framework;
8. Fair comparison of logistic regression, random forest, histogram gradient boosting, and artificial neural network models.

All comparisons used the same grouped-validation structure and primary performance criterion.

### 2.15. Repeated Household-Grouped Nested Cross-Validation

**Figure 3:**
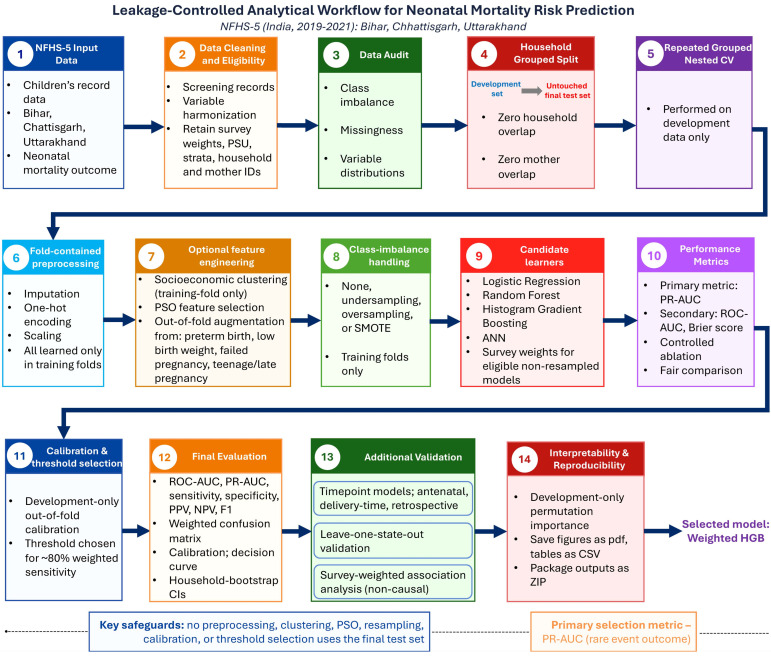
Leakage-controlled analytical workflow incorporating household-grouped partitioning, repeated nested cross-validation, fold-contained preprocessing, clustering, PSO, resampling, feature augmentation, model comparison, calibration, threshold selection, and final evaluation.

Model development used repeated household-grouped nested cross-validation. The outer folds estimated model performance, while the inner training process handled preprocessing, clustering, PSO feature selection, augmentation, resampling, and learner fitting. No information from the outer validation fold was used during any upstream modelling step. The same household grouping was maintained throughout the validation procedure. The primary model selection metric was precision–recall area under the curve because neonatal mortality was rare. The one-standard-error rule was applied to avoid selecting unnecessarily complex models when performance differences were small. A detailed workflow for the full mechanism is shown in Figure 3.

### 2.16. Probability Calibration and Screening-Threshold Selection

Calibration was based on household-grouped out-of-fold development predictions generated through the complete nested pipeline. A probability calibrator was fitted using development predictions only. The final classification threshold was selected from the calibrated development predictions to target approximately 80% survey-weighted sensitivity. Neither calibration nor threshold selection used the final-test dataset. The selected threshold was subsequently applied unchanged to the final-test probabilities.

### 2.17. Performance Evaluation

**Figure 4:**
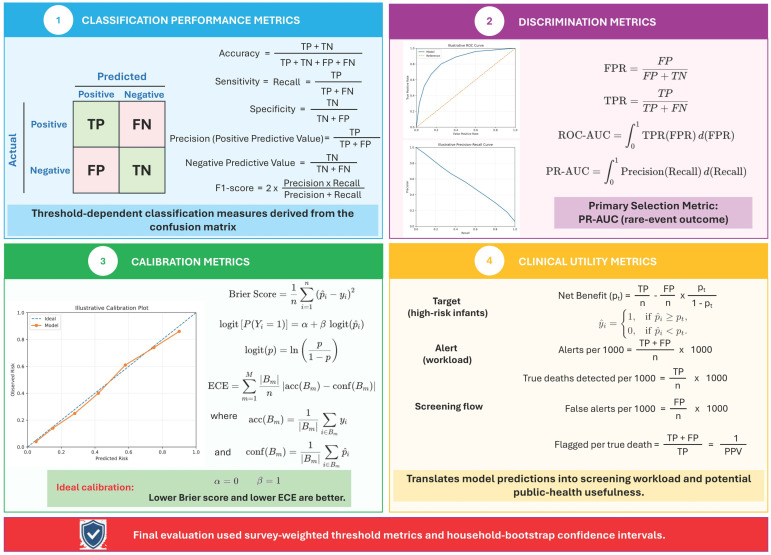
Evaluation framework for rare-event neonatal mortality prediction, including discrimination, threshold-dependent classification, calibration, and decision-curve measures.

Model discrimination was assessed using ROC-AUC and precision–recall AUC, with PR-AUC designated as the primary metric. Classification performance at the selected screening threshold was assessed using sensitivity, specificity, positive predictive value, negative predictive value, F1-score, and weighted confusion matrices. Probability accuracy and agreement were assessed using the Brier score, calibration intercept, calibration slope, expected calibration error, and calibration plots. Decision-curve analysis was used to estimate potential net benefit across clinically relevant probability thresholds. Operational screening burden was reported as alerts, true deaths detected, false alerts, and individuals flagged per true death detected per 1000 births. These metrics are displayed in Figure 4.

### 2.18. Statistical Uncertainty and Model Comparison

Uncertainty in final-test performance was quantified using household-grouped bootstrap resampling, thereby preserving within-household dependence. Ninety-five per cent confidence intervals were calculated for ROC-AUC, PR-AUC, sensitivity, specificity, positive and negative predictive values, F1-score, Brier score, calibration intercept, calibration slope, and expected calibration error. Differences between model specifications across repeated cross-validation folds were evaluated using corrected repeated cross-validation confidence intervals and tests that accounted for dependence created by overlapping training sets.

### 2.19. Leave-One-State-Out Validation

Geographic transportability was examined through leave-one-state-out validation. In each analysis, models were trained using data from two states and evaluated in the third state. Preprocessing, clustering, PSO, augmentation, calibration, and threshold selection were repeated using only the two-state training data. The held-out state was not used during model development. This analysis was interpreted as internal geographic validation rather than independent external validation.

### 2.20. Model Interpretation

Permutation importance was calculated using development-validation data rather than the final-test dataset. Importance values reflected the reduction in predictive performance when individual features were permuted. These values were interpreted as measures of predictive contribution and not as causal effects or evidence of modifiable risk factors.

### 2.21. Survey-Weighted Association Analysis

A separate survey-weighted penalised logistic regression was fitted using a prespecified reduced set of maternal, socioeconomic, pregnancy, and healthcare access variables. DHS sampling weights were incorporated, and uncertainty was estimated through stratified primary-sampling-unit bootstrap resampling. Regularisation was used to improve numerical stability in the presence of rare outcomes and sparse categories. The resulting coefficients and confidence intervals were interpreted as adjusted associations only. They were not used to establish mediation, treatment effects, or causal mechanisms.

### 2.22. Software and Reproducibility

The analysis was conducted in Python (v3.11) using pandas (v2.2.2), NumPy (v2.0.2), scikit-learn (v1.5.2), imbalanced-learn (v0.12.4), SciPy (v1.14.1), statsmodels (0.14.2), Matplotlib (v3.9.2), and related scientific-computing libraries. Random seeds were fixed for reproducibility. The fully executed notebook, fitted final model pipeline, reproducibility manifest, and selected machine-readable result and audit files are provided as Supplementary Materials. NFHS-5 individual-level microdata are not publicly distributed and must be obtained directly from the Demographic and Health Surveys Program under its applicable data-use conditions.

### 2.23. Ethical Considerations

The study used de-identified secondary data obtained through the Demographic and Health Surveys programme. No primary data were collected, and no participants were contacted by the authors. Access to the NFHS-5 microdata was subject to DHS data-use approval. The analysis therefore involved no additional intervention or direct risk to participants.

## 3. Results

### 3.1. Study Population and Analytical Integrity

The final analytical dataset included 33,338 child records from Bihar, Chhattisgarh, and Uttarakhand. Bihar contributed 21,040 records and 736 neonatal deaths, Chhattisgarh contributed 8514 records and 252 neonatal deaths, and Uttarakhand contributed 3784 records and 104 neonatal deaths. The survey-weighted neonatal mortality proportions were 3.43%, 3.06%, and 3.31%, respectively. Household-grouped partitioning allocated 26,652 observations to model development and 6686 observations to final testing. No household or mother appeared in both partitions. The detailed cohort distributions, survey identifiers, and split-integrity results are reported in Supplementary Tables S1–S3.

### 3.2. Overall Predictive Performance of the Selected Model

Histogram gradient boosting was selected as the final learner using repeated household-grouped nested cross-validation, with precision–recall area under the curve as the primary model selection metric. On the untouched final-test set, the model achieved a ROC-AUC of 0.755 (95% CI: 0.718–0.796) and a PR-AUC of 0.202 (95% CI: 0.138–0.267). At the screening threshold selected exclusively from fully nested out-of-fold development predictions, sensitivity was 0.813 (95% CI: 0.782–0.890), specificity was 0.517 (95% CI: 0.512–0.540), positive predictive value was 0.063 (95% CI: 0.052–0.074), and negative predictive value was 0.989 (95% CI: 0.984–0.992).

**Figure 5:**
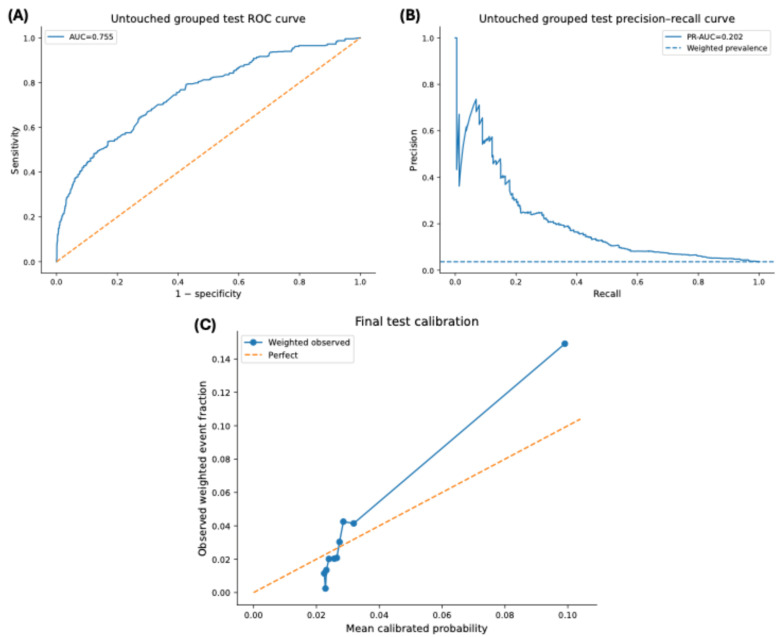
Discrimination and calibration of the selected weighted histogram gradient-boosting model on the untouched final-test set. (**A**) presents the receiver operating characteristic curve, (**B**) presents the precision–recall curve, and (**C**) presents the probability calibration curve.

The Brier score was 0.033 (95% CI: 0.028–0.038). The calibration intercept was 0.922 (95% CI: −0.158–2.058), the calibration slope was 1.242 (95% CI: 0.925–1.569), and the expected calibration error was 0.017 (95% CI: 0.012–0.022). Final-test discrimination and calibration are shown in Figure 5, while the complete set of final-test estimates and household-bootstrap confidence intervals is presented in Table 1.

### 3.3. Comparison of Candidate Learners

**Figure 6:**
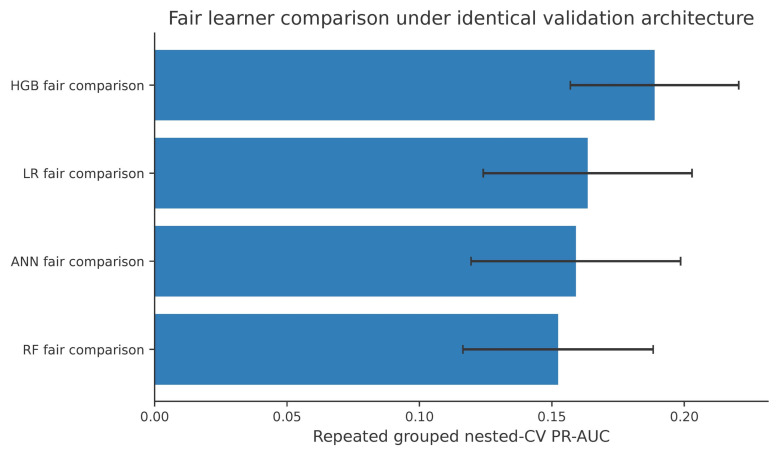
Fair comparison of candidate learners under repeated household-grouped cross-validation. All models were evaluated using the same grouped folds and PR-AUC as the primary metric.

Under identical repeated household-grouped validation, histogram gradient boosting achieved the highest mean PR-AUC of 0.189, followed by logistic regression at 0.164, artificial neural network at 0.159, and random forest at 0.152. The corresponding mean ROC-AUC values were 0.778, 0.757, 0.753, and 0.771, respectively. The corrected paired comparison between histogram gradient boosting and baseline logistic regression produced a mean PR-AUC difference of 0.037 (corrected 95% CI: 0.007–0.068; corrected *p* = 0.019). Figure 6 presents the comparative cross-validation performance, and Table 2 reports the numerical results.

### 3.4. Marginal Contribution of Framework Components

Baseline logistic regression achieved a mean PR-AUC of 0.151. Adding socioeconomic clustering produced a mean PR-AUC of 0.152, while PSO feature selection produced 0.161. The combination of clustering and PSO achieved 0.164. Adding SMOTE resulted in a mean PR-AUC of 0.162, and adding out-of-fold feature augmentation also resulted in 0.162. The complete logistic regression pipeline achieved 0.159.

**Figure 7:**
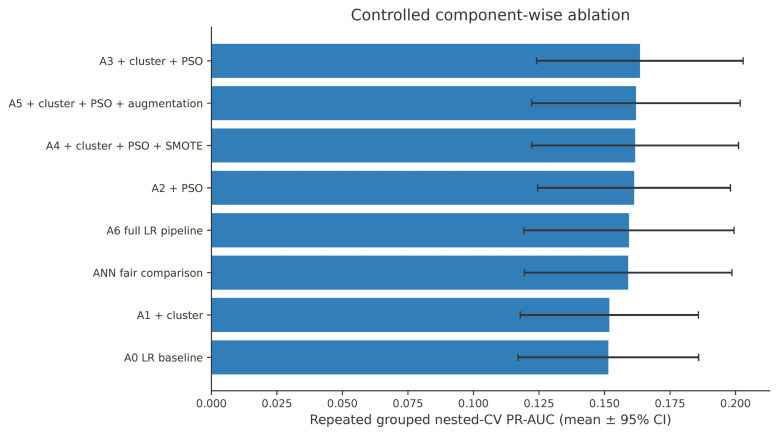
Controlled ablation of socioeconomic clustering, PSO feature selection, SMOTE, and out-of-fold feature augmentation. All specifications were evaluated using identical repeated household-grouped validation folds.

Relative to baseline logistic regression, none of the clustering, PSO, SMOTE, feature-augmentation, or complete-pipeline specifications had a corrected confidence interval that excluded zero. The ablation results are visualised in Figure 7 and reported numerically in Table 3.

### 3.5. Prediction Performance at Different Information Timepoints

**Figure 8:**
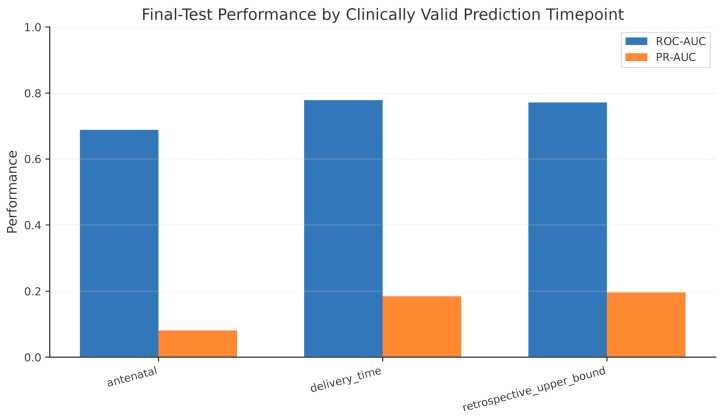
Final-test performance according to prediction timepoint. The antenatal model included variables available before delivery, the delivery time model added information available during or immediately after childbirth, and the retrospective model used the complete eligible feature set.

Prediction performance varied according to the time at which input variables became available. The antenatal model achieved a ROC-AUC of 0.688 and PR-AUC of 0.081. At its development-selected threshold, sensitivity was 0.802 and specificity was 0.423. The delivery time model achieved a ROC-AUC of 0.778 and PR-AUC of 0.184, with sensitivity of 0.846 and specificity of 0.545. The retrospective upper-bound model achieved a ROC-AUC of 0.771 and PR-AUC of 0.196, with sensitivity of 0.768 and specificity of 0.593. Figure 8 compares the three prediction settings, and Table 4 provides the complete estimates.

Across the three prediction time points, sensitivity ranged from 0.768 to 0.846, indicating that the model correctly identified approximately 77–85% of neonatal deaths. Although specificity was modest (0.423–0.593), the NPV remained consistently high (0.983–0.989). This high NPV is expected because neonatal death was a relatively infrequent event in the held-out dataset. Consequently, among neonates predicted to survive, the great majority were indeed alive, resulting in very few false-negative predictions relative to the large number of true negatives. Conversely, the PPV was low (0.050–0.067), reflecting the low prevalence of neonatal death and the relatively high number of false-positive predictions. Thus, despite reasonable sensitivity, many neonates predicted to die ultimately survived, which lowered the PPV. These findings are mathematically consistent with the observed class imbalance and do not indicate an inconsistency in the model’s performance metrics.

### 3.6. Geographic Transportability

**Figure 9:**
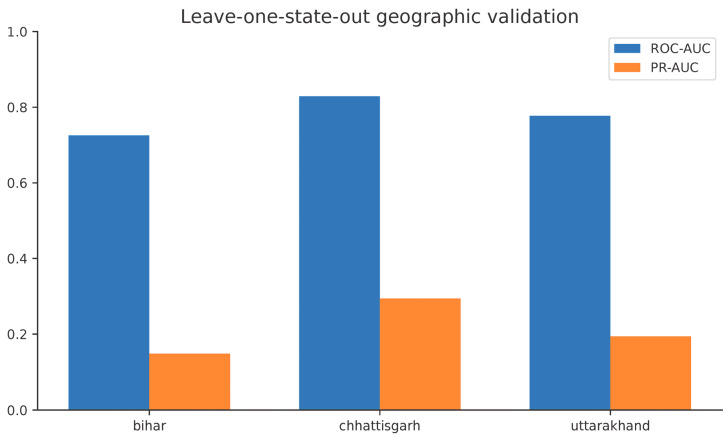
Leave-one-state-out validation across Bihar, Chhattisgarh, and Uttarakhand. Each held-out state was evaluated using a model developed, calibrated, and compared with threshold exclusively on the other two states.

Leave-one-state-out validation showed variation in performance across the three study states. When Bihar was held out, the model achieved a ROC-AUC of 0.731 and a PR-AUC of 0.147, with sensitivity of 0.949 and specificity of 0.241. When Chhattisgarh was held out, ROC-AUC was 0.823, and PR-AUC was 0.295, with sensitivity of 0.746 and specificity of 0.692. When Uttarakhand was held out, ROC-AUC was 0.786, and PR-AUC was 0.184, with sensitivity of 0.806 and specificity of 0.645. Figure 9 presents the state-wise comparison, and Table 5 provides the full results. Although the model’s sensitivity was 0.949, the negative predictive value (NPV) was 0.992. In our analysis, the outcome variable was coded as neonatal death = 1 and neonatal alive = 0. Therefore, sensitivity represents the proportion of neonatal deaths correctly identified by the model, whereas NPV represents the proportion of neonates predicted to survive (negative test result) who were indeed alive. NPV is influenced not only by sensitivity but also by specificity and the prevalence of neonatal death. Because neonatal death was relatively infrequent in our dataset, the number of true negatives (surviving neonates correctly classified) greatly exceeded the number of false negatives (deaths incorrectly classified as alive), resulting in a high NPV of 0.992, despite a sensitivity of 0.949.

### 3.7. Survey-Weighted Associations with Neonatal Mortality

**Figure 10:**
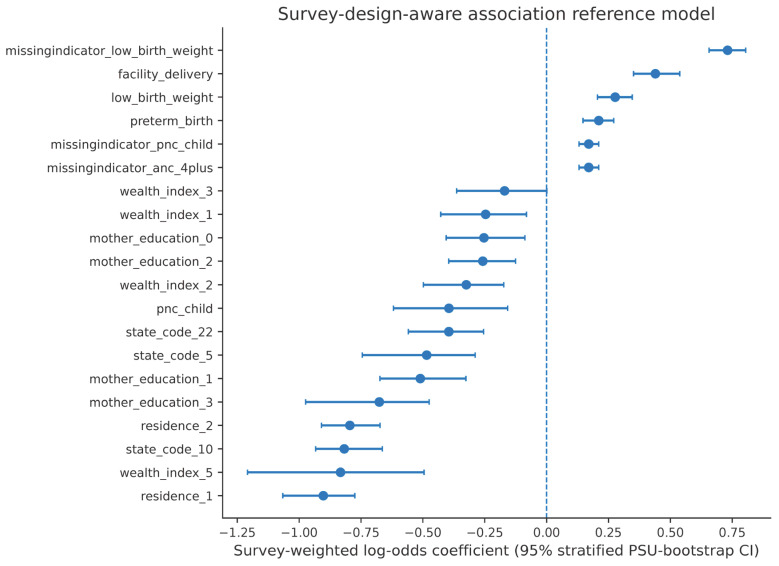
Survey-weighted adjusted associations with neonatal mortality. Points indicate odds ratios and horizontal lines indicate stratified PSU-bootstrap 95% confidence intervals. The estimates are associative and are not interpreted causally.

In the survey-weighted penalised logistic regression analysis, preterm birth was positively associated with neonatal mortality (OR = 1.235, 95% CI: 1.158–1.312), as was low birth weight (OR = 1.319, 95% CI: 1.228–1.414). Facility delivery was positively associated with the recorded outcome (OR = 1.552, 95% CI: 1.421–1.712), whereas postnatal care for the child was negatively associated with neonatal mortality (OR = 0.674, 95% CI: 0.539–0.855). The confidence intervals for teenage or late pregnancy, failed pregnancy history, four or more antenatal care visits, and marriage before 18 years included the null value. Figure 10 displays the adjusted odds ratios, and Table 6 provides the corresponding coefficients and uncertainty estimates.

## 4. Discussion

### 4.1. Principal Findings

This study developed and evaluated a leakage-controlled, household-grouped, and survey-aware framework for neonatal mortality prediction using NFHS-5 data from Bihar, Chhattisgarh, and Uttarakhand. The principal finding was that histogram gradient boosting provided the strongest overall predictive performance among the evaluated learners. On the untouched final-test set, the model achieved moderate discrimination, with a ROC-AUC of 0.755 and a PR-AUC of 0.202, while retaining high sensitivity and negative predictive value. However, the positive predictive value remained low, reflecting the rarity of neonatal death and the substantial number of false-positive alerts generated at the selected screening threshold. The controlled ablation analysis further showed that socioeconomic clustering, PSO feature selection, SMOTE, and out-of-fold feature augmentation provided little independent improvement beyond simpler specifications. Performance also varied according to the time at which predictor information became available and across the three held-out states. These findings indicate that the main contribution of the study lies in rigorous validation and transparent assessment of model utility rather than in the superiority of a complex augmented architecture.

### 4.2. Selected Model’s Predictive Performance

The selected histogram gradient-boosting model demonstrated moderate ability to distinguish neonatal deaths from survivors. The ROC-AUC indicated acceptable ranking performance, while the PR-AUC provided a more conservative and relevant assessment given the low prevalence of neonatal death. The difference between these two measures is important. The ROC-AUC can remain relatively favourable in highly imbalanced datasets because it is influenced strongly by the large number of correctly ranked negative observations. The PR-AUC, in contrast, reflects the balance between detecting neonatal deaths and avoiding false-positive alerts, and therefore provides a more realistic indication of performance for rare-event screening.

The high sensitivity suggests that the selected threshold identified most neonatal deaths in the final-test set. Similarly, the very high negative predictive value indicates that observations classified as low risk were unlikely to experience neonatal death. These properties may be useful in a preliminary screening context where the primary aim is to avoid missing potentially high-risk cases. However, the low positive predictive value means that most high-risk classifications were false-positive alerts. Consequently, the model should not be interpreted as a diagnostic tool, nor should its output independently trigger treatment, referral, or resource-intensive intervention. Its most plausible role would be to support low-cost secondary review, additional counselling, or closer observation within an existing maternal and neonatal care pathway.

The calibration results were broadly acceptable, although not ideal. The calibration slope was reasonably close to one, and the expected calibration error was low, indicating that predicted probabilities were generally aligned with observed outcomes. Nevertheless, the calibration intercept was imprecisely estimated, and the confidence interval included values consistent with some degree of systematic overprediction or underprediction. This reinforces the need for recalibration before implementation in settings that differ from the development population.

### 4.3. Candidate Learners–Comparisons

Histogram gradient boosting outperformed logistic regression, random forest, and the artificial neural network under the same household-grouped validation protocol. This finding is consistent with the strengths of gradient-boosting methods for structured tabular data. Such models can represent nonlinear relationships, threshold effects, and interactions without requiring extensive manual specification, while generally being less data-demanding than deep neural networks.

The artificial neural network did not outperform the simpler alternatives. This is an important result because neural networks are sometimes assumed to be inherently superior due to their complexity. In the present study, the combination of a moderately sized tabular dataset, heterogeneous categorical and continuous variables, a rare outcome, and substantial missingness likely favoured tree-based boosting over a neural architecture. Neural networks can require larger effective sample sizes, careful tuning, and more positive outcome events to achieve stable performance. Their flexibility may also increase variance when the number of events is limited.

Random forest achieved a ROC-AUC comparable to histogram gradient boosting but a lower PR-AUC. This suggests that it ranked positive and negative observations reasonably well overall but was less effective at concentrating true neonatal deaths among the highest-risk predictions. Logistic regression provided a useful baseline and retained interpretability, but its linear specification was less able to capture the nonlinear patterns and interactions identified by boosting. The comparison therefore supports the use of histogram gradient boosting as the final predictive learner while also demonstrating why fair evaluation against simpler models is necessary.

### 4.4. Contribution of Clustering, PSO, SMOTE, and Feature Augmentation

The ablation analysis showed that the individual framework components contributed little additional predictive value. Socioeconomic clustering produced a negligible change in PR-AUC, suggesting that the original state, residence, wealth, and education variables already contained most of the information represented by the derived cluster assignments. Clustering may still offer descriptive or policy-oriented value by summarising socioeconomic profiles, but its contribution to predictive accuracy was minimal.

PSO feature selection produced a small numerical improvement, but the corrected confidence interval included zero. This finding suggests that PSO may have removed some redundant predictors without consistently improving out-of-sample performance. The result also illustrates that metaheuristic feature selection should not be assumed to improve prediction merely because it reduces dimensionality. Its value depends on whether the selected subset remains stable across folds and whether the removed variables are truly uninformative.

SMOTE did not improve performance after clustering and PSO. This result is plausible because synthetic oversampling can alter the empirical joint distribution of survey variables and may generate observations that do not correspond closely to realistic maternal or neonatal profiles. In addition, synthetic samples cannot be assigned natural DHS sampling weights. The limited benefit of SMOTE in this study suggests that survey-weighted non-resampled models may be preferable when the learner can already accommodate class imbalance.

Out-of-fold feature augmentation also failed to produce a clear gain. Although the predicted probabilities of preterm birth, low birth weight, failed pregnancy, and teenage or late pregnancy were generated without leakage, these intermediate predictions may not have added substantial information beyond the original variables. Their first-stage prediction errors may also have propagated into the final classifier. Overall, the ablation findings demonstrate that combining more components does not necessarily produce a better model. The strongest improvement came from the choice of learner rather than from additional clustering, feature selection, resampling, or augmentation.

### 4.5. Prediction at Antenatal, Delivery Time, and Retrospective Stages

The prediction-timepoint analysis showed that antenatal performance was notably weaker than delivery time and retrospective performance. This difference is clinically important because antenatal prediction is the most attractive setting for preventive action, yet it relies on a more restricted set of variables. Maternal characteristics, socioeconomic conditions, obstetric history, and antenatal care indicators provided some discriminatory information, but they were insufficient for strong rare-event prediction. Performance improved when delivery time variables became available. Gestational duration, birth weight, delivery setting, and related information are closely associated with neonatal survival and therefore increased discrimination. The delivery time model achieved performance close to the retrospective upper-bound model, suggesting that most of the useful predictive information was available by the time of childbirth or immediately thereafter. The retrospective model achieved the highest PR-AUC but should not be interpreted as an early-warning system because it included postnatal information. Its role is best understood as an upper-bound benchmark that quantifies the maximum performance achievable using the complete NFHS-5 feature set. These findings indicate that a practical implementation should distinguish clearly between antenatal risk screening and delivery time neonatal risk assessment. A delivery time model may be the more realistic candidate for initial operational testing, while antenatal prediction would require additional data sources or biomarkers to achieve stronger performance.

### 4.6. Cross-Geography Applicability

Leave-one-state-out validation demonstrated substantial heterogeneity in performance across Bihar, Chhattisgarh, and Uttarakhand. The strongest performance was observed when Chhattisgarh was held out, whereas the Bihar analysis showed particularly low specificity despite very high sensitivity. This indicates that the model generated a large number of false-positive alerts in Bihar. Several factors may explain this variation. The states differ in population size, socioeconomic composition, healthcare access, maternal characteristics, and outcome prevalence. Bihar also contributed the largest proportion of the analytical dataset and excluding it from the model development may have altered both the distribution of predictors and the calibration of predicted risks. The observed heterogeneity suggests that a single threshold is unlikely to perform equally well across all settings. These results do not demonstrate broad external generalisability. Rather, they provide internal geographic validation across three states. Before implementation, the model would require evaluation in additional Indian states and in more recent routine or facility-based data. State-specific recalibration and threshold selection may also be necessary to achieve an acceptable balance between sensitivity and false-positive burden.

### 4.7. Survey-Weighted Association Analysis

The survey-weighted association analysis identified positive associations between neonatal mortality and preterm birth, low birth weight, and facility delivery, while postnatal care for the child was negatively associated with mortality. The associations with preterm birth and low birth weight are consistent with their established roles as major indicators of neonatal vulnerability. Both conditions reflect biological immaturity, impaired foetal growth, and increased susceptibility to respiratory, other infectious, and metabolic complications. The positive association with facility delivery should not be interpreted as evidence that facility delivery increases neonatal mortality. Women with severe complications, high-risk pregnancies, obstructed labour, or foetal distress are more likely to deliver in health facilities. This process of referral and severity-related selection can create a positive association even when facility-based care is beneficial. The finding therefore likely reflects residual confounding by clinical severity and referral patterns.

The negative association with postnatal care is compatible with the role of early neonatal assessment in identifying complications and supporting timely management. However, the observational design prevents causal interpretation, and postnatal care uptake may also be associated with unmeasured factors such as maternal health literacy, facility access, and socioeconomic advantage. Other variables, including teenage or late pregnancy, failed pregnancy history, four or more antenatal care visits, and marriage before 18 years, had confidence intervals that included the null value after adjustment. Their lack of statistical precision does not imply that they are clinically unimportant. Rather, the associations may be mediated, confounded, measured imprecisely, or heterogeneous across subgroups. The regression findings should therefore be interpreted as adjusted associations within this specific survey population.

### 4.8. Implications for Neonatal Risk Screening and Decision Support

The findings support the potential use of machine learning as one component of neonatal risk screening but do not establish a deployable decision-support system. The selected model could theoretically be integrated into an HMIS or community health worker application to flag records for additional review. However, the low positive predictive value means that such integration would generate many false alerts. The downstream action must therefore be inexpensive, non-invasive, and unlikely to cause harm. A plausible workflow would involve automatic risk scoring at delivery, followed by human review of flagged cases, confirmation of key clinical information, and targeted observation or counselling. The model should not independently determine admission, treatment, or referral. Prospective implementation would also require monitoring of calibration drift, threshold performance, subgroup fairness, workflow burden, and unintended consequences.

### 4.9. Strengths of the Study

The study has several methodological strengths. Household-grouped splitting prevented records from the same household or mother from appearing in both development and test datasets. Repeated grouped nested cross-validation ensured that preprocessing, clustering, PSO, feature augmentation, resampling, calibration, and threshold selection were performed within the appropriate training data. The final-test set remained untouched until final evaluation. The study prioritised PR-AUC and reported a comprehensive set of rare-event metrics, including calibration and clinical utility measures. Household-grouped bootstrap confidence intervals quantified uncertainty while preserving within-household dependence. Controlled ablation allowed the contribution of each framework component to be assessed directly. Prediction timepoint analysis clarified the distinction between antenatal, delivery time, and retrospective use, while leave-one-state-out validation assessed geographic transportability. The survey-weighted association analysis also incorporated DHS weights, strata, and PSUs and was kept conceptually separate from predictive modelling.

### 4.10. Limitations

Several limitations should be acknowledged. First, NFHS-5 is an observational survey, and many variables rely on maternal recall or categorical reporting. Misclassification and recall error may therefore affect both predictors and outcomes. Second, neonatal death was rare, resulting in limited positive predictive value and wide uncertainty for some metrics. Third, although the model was evaluated using rigorous internal and geographic validation, no independent external dataset was available. The results therefore cannot establish generalizability to other Indian states, countries, time periods, or routine HMIS data. Fourth, antenatal prediction remained weak, limiting the usefulness of the model for early preventive intervention. Fifth, survey weights could be used directly in eligible non-resampled models, but synthetic resampling methods could not naturally preserve the complex survey design. Sixth, the penalised survey-weighted regression provided stable association estimates but is not equivalent to a conventional unpenalised design-based causal model. Seventh, households from the same PSU could appear across development and test partitions, leaving some potential contextual dependence. Finally, the study did not evaluate prospective implementation, cost-effectiveness, fairness across demographic groups, alert fatigue, workflow integration, or the effects on neonatal outcomes. These issues must be addressed before practical deployment.

### 4.11. Future Research

Future studies should validate the selected model prospectively using routine HMIS, facility, or community health worker data. Temporal validation and evaluation in additional Indian states are needed to assess transportability. State-specific recalibration and threshold selection should be examined, particularly for settings such as Bihar where specificity was low. Further work should also assess fairness across socioeconomic, geographic, ethnic, and maternal subgroups; evaluate the cost and workload associated with false-positive alerts; and involve healthcare workers in human-factors and usability testing. Additional predictors available in clinical settings, including laboratory measurements, physiological observations, and non-invasive approaches such as infant cry acoustic analysis, may improve antenatal or early neonatal prediction. Any future DSS should be evaluated prospectively for safety, clinical utility, and impact on service delivery before large-scale implementation.

## 5. Conclusions

This study developed and evaluated a household-grouped, leakage-controlled, and survey-aware framework for neonatal mortality risk prediction using NFHS-5 data from Bihar, Chhattisgarh, and Uttarakhand. Histogram gradient boosting achieved the strongest overall performance among the evaluated learners, with moderate discrimination, acceptable calibration, high sensitivity, and a very high negative predictive value on the untouched final-test set. However, the low positive predictive value indicated a substantial false-positive burden, limiting the model’s immediate use to low-cost risk screening rather than diagnosis, automatic referral, or treatment decision-making. The controlled ablation analysis showed that socioeconomic clustering, PSO feature selection, SMOTE, and out-of-fold feature augmentation provided little additional predictive benefit. Performance depended more strongly on the selected learner and on the time at which information became available. Delivery time prediction was more informative than antenatal prediction, while geographic validation showed meaningful variation across the three states, particularly in specificity. These findings support further development of a neonatal risk-screening framework but do not demonstrate causal effects, improved neonatal outcomes, or a fully implemented decision-support system. Prospective validation in routine clinical or HMIS settings, external evaluation across additional populations, state-specific recalibration, and assessment of workflow burden, fairness, safety, and cost-effectiveness are required before operational deployment.

## Figures and Tables

**Table 1:** Final-test performance of the selected weighted histogram gradient-boosting model.

Metric	Estimate	95% CI
ROC-AUC	0.755	0.718–0.796
PR-AUC	0.202	0.138–0.267
Sensitivity	0.813	0.782–0.890
Specificity	0.517	0.512–0.540
Positive predictive value	0.063	0.052–0.074
Negative predictive value	0.989	0.984–0.992
F1-score	0.117	0.098–0.136
Brier score	0.033	0.028–0.038
Calibration intercept	0.922	−0.158–2.058
Calibration slope	1.242	0.925–1.569
Expected calibration error	0.017	0.012–0.022

Note: Confidence intervals were obtained from 1000 household-grouped bootstrap replicates.

**Table 2:** Repeated household-grouped cross-validation performance of candidate learners.

Learner	Folds	Mean PR-AUC	SD	Corrected 95% CI	Mean ROC-AUC	Mean Brier Score
Logistic regression	15	0.164	0.036	0.124–0.203	0.757	0.193
Random forest	15	0.152	0.033	0.116–0.188	0.771	0.057
Histogram gradient boosting	15	0.189	0.029	0.157–0.221	0.778	0.029
Artificial neural network	15	0.159	0.036	0.119–0.199	0.753	0.030

Note: Confidence intervals use the corrected repeated cross-validation standard error. Histogram gradient boosting versus baseline logistic regression: mean PR-AUC difference = 0.037, corrected 95% CI = 0.007–0.068, corrected *p* = 0.019.

**Table 3:** Corrected paired differences in PR-AUC relative to baseline logistic regression.

Specification	Mean PR-AUC Difference	Corrected SE	Corrected 95% CI	Corrected *p*-Value
Clustering	0.0004	0.0017	−0.0032–0.0040	0.820
PSO feature selection	0.0098	0.0127	−0.0173–0.0369	0.451
Clustering + PSO	0.0120	0.0105	−0.0106–0.0346	0.272
Clustering + PSO + SMOTE	0.0102	0.0108	−0.0129–0.0334	0.360
Clustering + PSO + augmentation	0.0105	0.0110	−0.0131–0.0342	0.356
Complete LR pipeline	0.0079	0.0115	−0.0169–0.0326	0.506

Note: Differences were evaluated using the Nadeau–Bengio corrected repeated k-fold comparison.

**Table 4:** Final-test performance according to prediction timepoint.

Prediction Timepoint	Features	Threshold	ROC-AUC	PR-AUC	Sensitivity	Specificity	PPV	NPV	Brier Score
Antenatal	36	0.0292	0.688	0.081	0.802	0.423	0.050	0.983	0.035
Delivery time	44	0.0260	0.778	0.184	0.846	0.545	0.066	0.989	0.033
Retrospective upper bound	46	0.0262	0.771	0.196	0.768	0.593	0.067	0.985	0.033

**Table 5:** Leave-one-state-out validation results.

Held-Out State	n	Deaths	Threshold	ROC-AUC	PR-AUC	Sensitivity	Specificity	PPV	NPV	Brier Score
Bihar	21,040	736	0.0257	0.731	0.147	0.949	0.241	0.043	0.992	0.031
Chhattisgarh	8514	252	0.0273	0.823	0.295	0.746	0.692	0.071	0.989	0.027
Uttarakhand	3784	104	0.0271	0.786	0.184	0.806	0.645	0.072	0.990	0.031

**Table 6:** Survey-weighted adjusted associations between prespecified variables and neonatal mortality.

Predictor	Coefficient	Bootstrap SE	Odds Ratio	95% CI
Preterm birth	0.211	0.034	1.235	1.158–1.312
Low birth weight	0.277	0.035	1.319	1.228–1.414
Teenage or late pregnancy	0.032	0.047	1.033	0.944–1.127
Failed pregnancy history	0.017	0.035	1.017	0.944–1.081
Four or more ANC visits	0.072	0.061	1.074	0.947–1.210
Facility delivery	0.440	0.046	1.552	1.421–1.712
Postnatal care for the child	−0.395	0.119	0.674	0.539–0.855
Married before 18 years	−0.085	0.047	0.918	0.836–1.006

Note: Estimates were obtained from sampling-weighted L2-penalised logistic regression with stratified PSU-bootstrap percentile intervals. The estimates describe adjusted associations and not causal effects.

## Data Availability

The data that support the findings of this study are available from: [https://dhsprogram.com/data/available-datasets.cfm] (accessed on 15 August 2024).

## Supplementary Materials

The following supporting information can be downloaded at https://www.mdpi.com/article/10.3390/healthcare14142144/s1, Table S1: State-specific cohort size and neonatal mortality distribution; Table S2: Survey and grouping identifier audit; Table S3: Household-grouped development and final-test split; Table S4: Complete ablation and learner-comparison summary; Table S5: Development-only screening-threshold selection; Table S6: Survey-weighted final-test confusion matrix; Table S7: Operational screening burden per 1000 births; Table S8: Weighted final-test calibration bins; Table S9: Survey-weighted association-model diagnostics; Figure S1: Distribution of neonatal survival and neonatal death in the analytical dataset; Figure S2: Missingness proportions for eligible model predictors; Figure S3: Correlation structure among selected numerical and encoded predictors; Figure S4: Survey-weighted confusion matrix for the selected final-test screening threshold; Figure S5: Decision-curve analysis of the selected model across candidate risk thresholds; Figure S6: Development-validation permutation importance for the selected model; Data File S1: repeated_grouped_nested_cv_fold_results.csv, fold-level results from repeated household-grouped nested cross-validation; Data File S2: final_development_pso_convergence.csv, convergence history from the final development data particle swarm optimisation; Data File S3: nested_pso_convergence_all_folds.csv, particle swarm optimisation convergence results across nested-validation folds; Data File S4: final_selected_features.csv, final predictor set selected during model development; Data File S5: development_validation_permutation_importance.csv, permutation importance results obtained from development-validation data; Data File S6: survey_weighted_stratified_psu_bootstrap_logistic_coefficients.csv, survey-weighted association-model coefficients and stratified primary sampling unit bootstrap results; Data File S7: reviewer_technical_compliance_audit.csv, technical-compliance and leakage-control audit; Data File S8: fully_nested_calibration_fold_audit.csv, fold-level audit of the fully nested calibration procedure; Data File S9: nested_first_stage_oof_audit.csv, audit of the nested out-of-fold first-stage prediction procedure; Data File S10: timepoint_antenatal_fully_nested_fold_audit.csv, fold-level audit of the antenatal prediction analysis; Data File S11: timepoint_delivery_time_fully_nested_fold_audit.csv, fold-level audit of the delivery time prediction analysis; Data File S12: timepoint_retrospective_upper_bound_fully_nested_fold_audit.csv, fold-level audit of the retrospective upper-bound analysis; Data File S13: loso_bihar_fully_nested_fold_audit.csv, fully nested leave-one-state-out audit with Bihar held out; Data File S14: loso_chhattisgarh_fully_nested_fold_audit.csv, fully nested leave-one-state-out audit with Chhattisgarh held out; Data File S15: loso_uttarakhand_fully_nested_fold_audit.csv, fully nested leave-one-state-out audit with Uttarakhand held out; Code File S1: MDPI Healthcare Final Executed Notebook.ipynb, fully executed notebook containing the analytical workflow; Model File S1: final_model_pipeline.joblib, fitted final model pipeline; Reproducibility File S1: reproducibility_manifest.json, software, parameter, random-seed, and execution manifest; Reproducibility File S2: output_file_inventory.csv, inventory of the supplied supplementary and reproducibility files.
