# Supplementary material for: Leakage-Controlled and Survey-Weighted Machine Learning for Neonatal Mortality Risk Prediction Using NFHS-5 Data
Source: Healthcare (Basel). 2026 Jul 16;14(14):2144. doi: 10.3390/healthcare14142144 (PMC13410558; doi:10.3390/healthcare14142144)
Supplement: Supplementary file 1 [file healthcare-14-02144-s001.zip › Supplementary_Materials_Healthcare_4268889.pdf]

Leakage-Controlled and Survey-Weighted Machine Learning for Neonatal Mortality Risk Prediction Using NFHS-5 Data

Moumita Mukherjee, Talha Ali Khan, and Raja Hashim Ali

Manuscript ID: healthcare-4268889

Overview. This document provides supplementary cohort, survey-design, partition-integrity, model-comparison, threshold-selection, calibration, operational-burden, and association-diagnostic results supporting the main manuscript. It also presents six supplementary figures describing outcome imbalance, missingness, predictor correlation, threshold-dependent classification, decision-curve analysis, and development-validation permutation importance. All results are derived from the leakage-controlled and household-grouped analytical workflow described in the manuscript.

Supplementary Tables

State-level cohort composition provides context for both the unweighted sample distribution and the survey-weighted outcome burden. Table S1 reports the number of children and neonatal deaths in each state together with the corresponding unweighted and survey-weighted mortality proportions, allowing differences in sample contribution to be distinguished from differences in weighted outcome prevalence.

Table S1: State-specific cohort size and neonatal mortality distribution

State	Unweighted n	Neonatal deaths	Unweighted mortality proportion	Survey-weighted mortality proportion
Bihar	21,040	736	0.0350	0.0343
Chhattisgarh	8,514	252	0.0296	0.0306
Uttarakhand	3,784	104	0.0275	0.0331

Note: *Mortality proportions are reported as proportions rather than percentages. Survey-weighted estimates use the normalized NFHS-5/DHS sampling weights retained for the analysis.*

The leakage-control and survey-aware analyses depended on complete identifiers for households, mothers, sampling weights, primary sampling units, survey strata, and states. Table S2 verifies the availability and completeness of these fields and reports the number of distinct non-missing values used to define grouping and survey structure.

Table S2: Survey and grouping identifier audit

Variable	Present	Non-missing observations	Unique non-missing values	Missing fraction
Household ID	Yes	33,338	22,097	0.000
Mother ID	Yes	33,338	23,366	0.000
Sample weight	Yes	33,338	3,373	0.000
PSU	Yes	33,338	3,402	0.000
Strata	Yes	33,338	312	0.000
State	Yes	33,338	3	0.000

Note: *PSU denotes primary sampling unit. A missing fraction of 0.000 indicates that the identifier was complete for all 33,338 analytical records.*

Partition integrity was examined separately by state to ensure that the household-grouped development and final-test split retained outcome representation without sharing related observations across partitions. Table S3 reports split-specific sample sizes, event rates, and overlap checks; zero mother and household overlap confirms that the grouping constraint was preserved.

Table S3: Household-grouped development and final-test split

Split	State	Children	Mothers	Deaths	Event rate	Weighted event rate	Mother overlap	Household overlap
Development	Bihar	16,828	11,084	585	0.0348	0.0337	0	0
Development	Chhattisgarh	6,796	5,230	200	0.0294	0.0297	0	0
Development	Uttarakhand	3,028	2,369	78	0.0258	0.0311	0	0
Final test	Bihar	4,212	2,790	151	0.0359	0.0367	0	0
Final test	Chhattisgarh	1,718	1,296	52	0.0303	0.0342	0	0
Final test	Uttarakhand	756	597	26	0.0344	0.0413	0	0

Note: *Event rate is the unweighted neonatal mortality proportion. Weighted event rate uses the retained sampling weights. Overlap values count identifiers appearing in both the development and final-test partitions.*

The complete ablation and fair learner comparison was conducted over the same 15 repeated household-grouped validation folds. Table S4 summarizes discrimination and probability-error measures for the baseline logistic-regression model, sequential framework components, and the four fairly compared learner families, with PR-AUC serving as the primary model-selection criterion.

Table S4: Complete ablation and learner-comparison summary

Specification	Folds	Mean PR-AUC	SD	Mean ROC-AUC	Mean Brier score
Baseline LR	15	0.151	0.031	0.758	0.186
LR + clustering	15	0.152	0.031	0.758	0.186
LR + PSO	15	0.161	0.033	0.756	0.195
LR + clustering + PSO	15	0.164	0.036	0.757	0.193
LR + clustering + PSO + SMOTE	15	0.162	0.036	0.756	0.203
LR + clustering + PSO + augmentation	15	0.162	0.036	0.757	0.193
Complete LR pipeline	15	0.159	0.036	0.756	0.202
Fair-comparison LR	15	0.164	0.036	0.757	0.193
Fair-comparison RF	15	0.152	0.033	0.771	0.057
Fair-comparison HGB	15	0.189	0.029	0.778	0.029
Fair-comparison ANN	15	0.159	0.036	0.753	0.030

Note: *LR, logistic regression; PSO, particle swarm optimisation; SMOTE, synthetic minority oversampling technique; RF, random forest; HGB, histogram gradient boosting; ANN, artificial neural network; PR-AUC, area under the precision-recall curve; ROC-AUC, area under the receiver-operating-characteristic curve. Values are means across 15 repeated grouped validation folds unless otherwise stated.*

The operating threshold was selected without reference to the untouched final-test set. Table S5 documents the development-only threshold-selection dataset, the prespecified sensitivity target, the selected probability threshold, and the resulting weighted performance calculated from fully nested household-grouped out-of-fold predictions.

Table S5: Development-only screening-threshold selection

Selection data	Target sensitivity	Selected threshold	ROC-AUC	PR-AUC	Sensitivity	Specificity	PPV	NPV
Fully nested household-grouped OOF development predictions	0.800	0.0264	0.777	0.180	0.800	0.564	0.059	0.988

Note: *OOF denotes out-of-fold. PPV, positive predictive value; NPV, negative predictive value. The selected threshold was subsequently applied unchanged to the final-test probabilities.*

The final-test classification results can be expressed as survey-weighted cell counts at the development-selected screening threshold. Table S6 separates surviving children and neonatal deaths by predicted category and provides the numerical values underlying the supplementary confusion-matrix figure.

Table S6: Survey-weighted final-test confusion matrix

Actual outcome	Predicted survival	Predicted high risk
Survival	3,342.54	3,123.63
Neonatal death	45.98	199.52

Note: *Because sampling weights were applied, the cell totals are weighted counts and are therefore not restricted to integer values.*

To translate threshold performance into an operationally interpretable screening burden, the final-test results were standardized per 1,000 births. Table S7 reports the expected number of alerts, detected deaths, false alerts, and individuals flagged for each true neonatal death detected at the selected threshold.

Table S7: Operational screening burden per 1,000 births

Threshold	Alerts per 1,000 births	True deaths detected per 1,000 births	False alerts per 1,000 births	Number flagged per true death detected
0.0264	487.65	30.75	456.90	15.86

Note: *The estimated burden supports interpretation as a low-cost screening aid only. The model is not proposed as a diagnostic system or as an automatic referral mechanism.*

Calibration was examined by grouping final-test predictions into ten probability bins and comparing average predicted risk with the observed survey-weighted outcome proportion. Table S8 provides the bin-level data used to assess whether predicted probabilities increased consistently with the observed frequency of neonatal death.

Table S8: Weighted final-test calibration bins

Bin	n	Mean predicted probability	Observed outcome proportion
1	706	0.0225	0.0115
2	779	0.0229	0.0024
3	537	0.0232	0.0135
4	658	0.0240	0.0201
5	726	0.0257	0.0203
6	606	0.0265	0.0208
7	680	0.0273	0.0303
8	657	0.0286	0.0425
9	668	0.0319	0.0415
10	669	0.0991	0.1492

Note: *Bins are ordered from the lowest to the highest predicted risk. The column n reports the unweighted number of records in each bin, whereas the observed outcome proportion is survey weighted.*

The separate association analysis was evaluated for survey-structure coverage, bootstrap completion, and coefficient stability. Table S9 confirms that all requested stratified PSU-bootstrap replicates were valid and that the penalised survey-weighted model produced finite coefficients across the prespecified diagnostic checks.

Table S9: Survey-weighted association-model diagnostics

Records	PSUs	Strata	Bootstrap replicates requested	Valid replicates	All coefficients finite	Maximum absolute coefficient
33,338	3,402	312	250	250	Yes	0.902

Note: *The association model used sampling-weighted L2-penalised logistic regression with stratified primary-sampling-unit bootstrap percentile intervals. These diagnostics support numerical stability but do not convert the observational associations into causal effects.*

Supplementary Figures

Neonatal mortality was a rare outcome in the analytical cohort, making accuracy alone an unsuitable basis for model assessment. Figure S1 visualizes the marked class imbalance that motivated the use of PR-AUC, threshold-specific predictive values, calibration assessment, and uncertainty estimates.

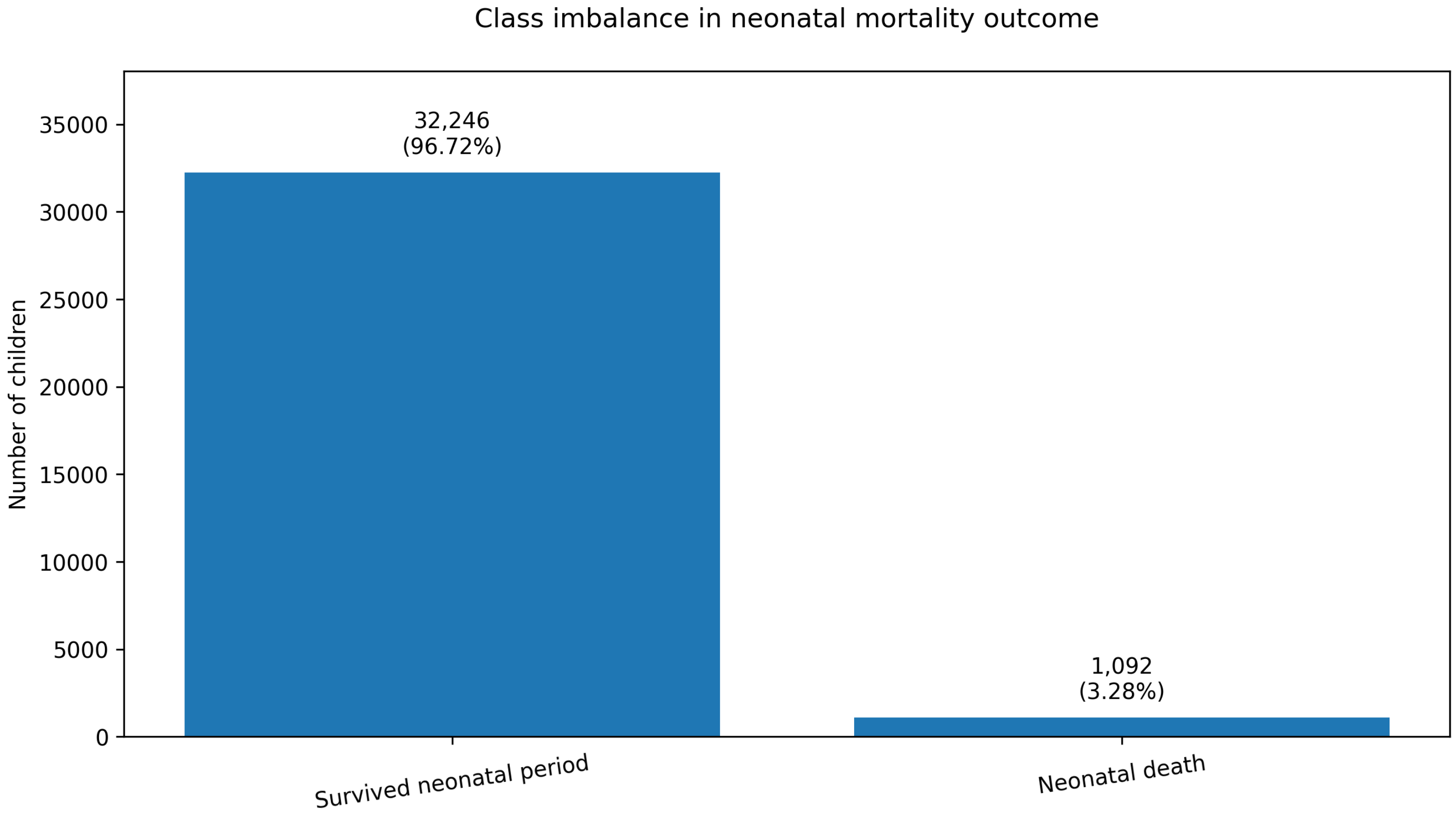

Figure S1: Distribution of neonatal survival and neonatal death in the analytical dataset.

Predictor missingness was audited before modelling to identify variables requiring fold-contained imputation and to distinguish genuinely sparse fields from complete predictors. Figure S2 ranks the eligible features by missing proportion and shows that missingness was concentrated in a limited subset of household, partner, assistance, and birth-history variables.

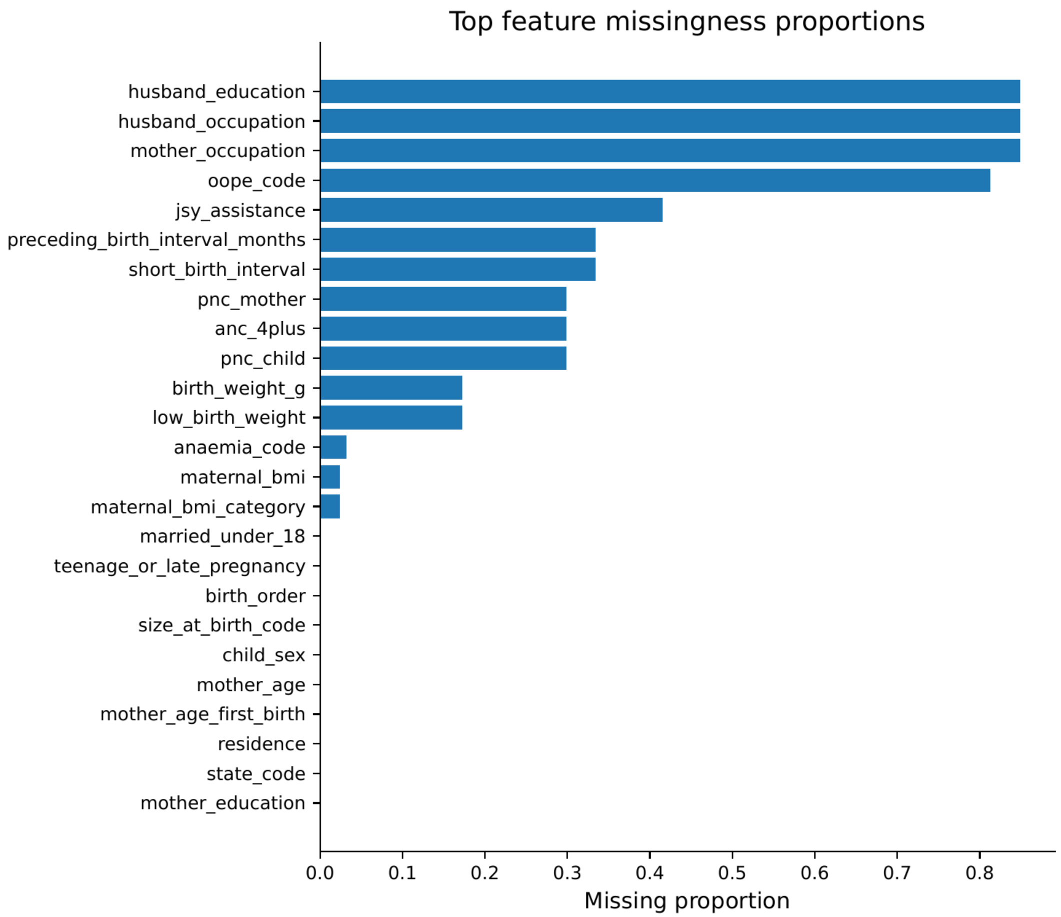

Figure S2: Missingness proportions for eligible model predictors.

Pairwise correlation analysis was used as a descriptive audit of redundancy and linear dependence among selected outcome-relevant numerical and encoded variables. Figure S3 displays the resulting correlation structure; the values are descriptive and should not be interpreted as causal relationships.

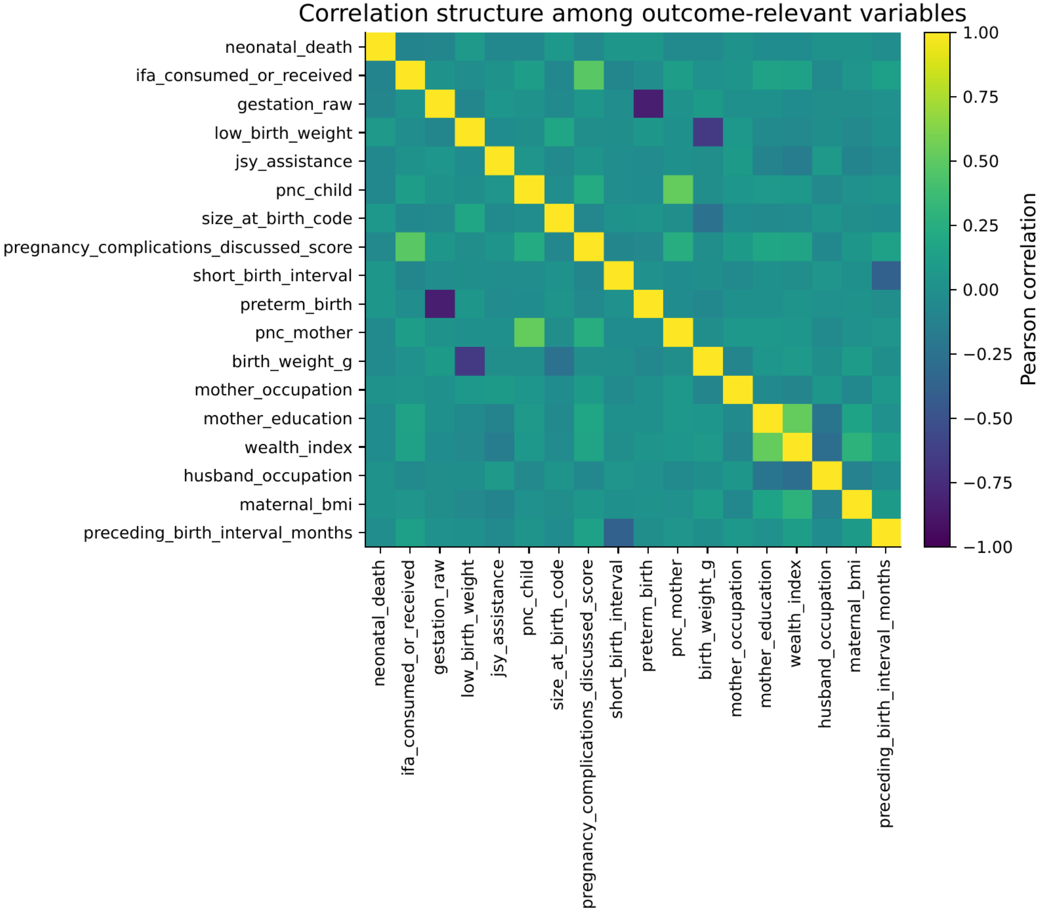

Figure S3: Correlation structure among selected numerical and encoded predictors.

The final-test confusion matrix provides a direct view of the trade-off created by the sensitivity-oriented screening threshold. Figure S4 shows that most neonatal deaths were flagged as high risk, while the large number of surviving children classified as high risk explains the low positive predictive value and substantial false-alert burden.

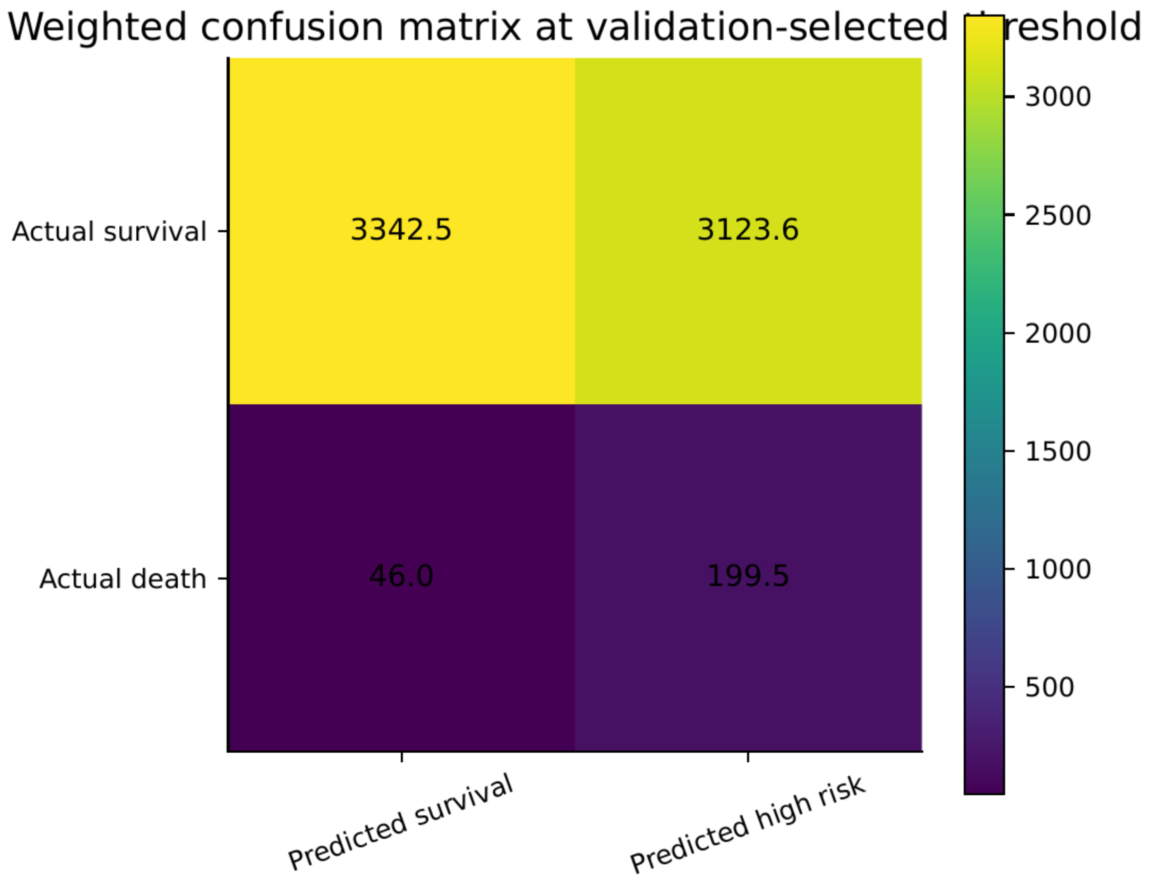

Figure S4: Survey-weighted confusion matrix for the selected final-test screening threshold.

Decision-curve analysis was used to compare the model with hypothetical treat-all and treat-none strategies across candidate probability thresholds. Figure S5 presents net benefit as a function of threshold and marks the development-selected operating point, supporting interpretation of potential utility only within low-cost screening contexts.

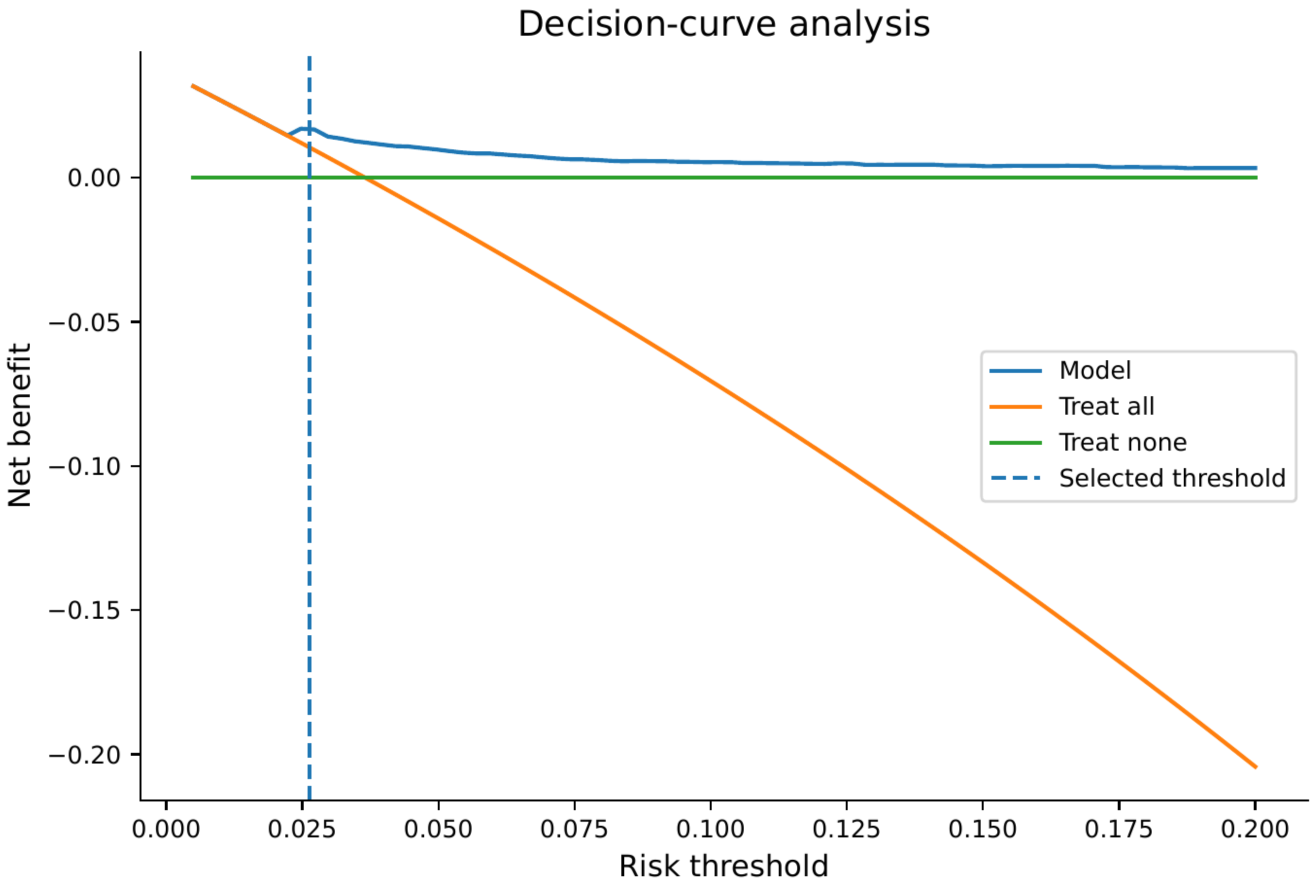

Figure S5: Decision-curve analysis of the selected model across candidate risk thresholds.

Model interpretation was performed on development-validation data rather than on the untouched final-test set. Figure S6 ranks features by the decrease in validation PR-AUC after permutation and displays uncertainty across the evaluation repeats; these values quantify predictive contribution and do not establish causal importance.

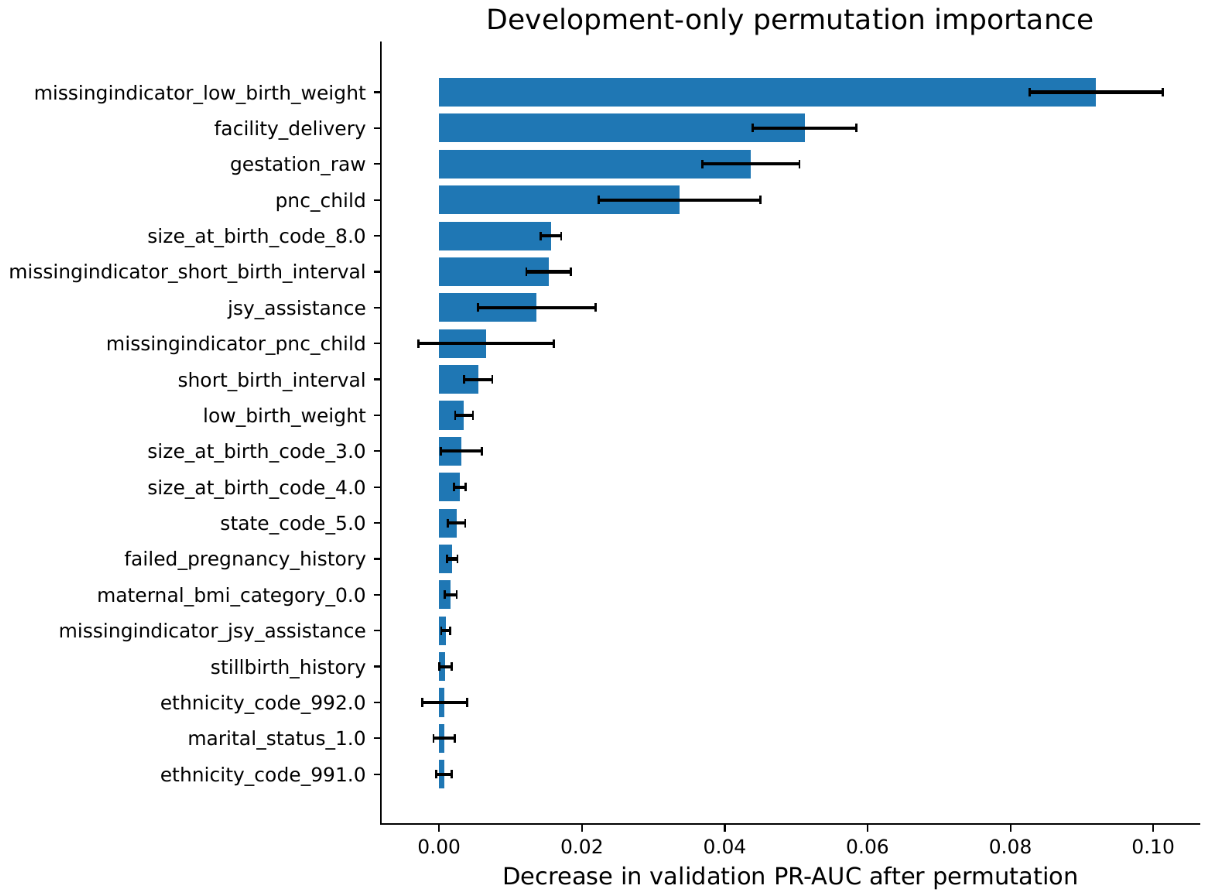

Figure S6: Development-validation permutation importance for the selected model.

Supplementary file note. The tables and figures in this document are intended for online publication alongside the article. Machine-readable CSV outputs, the executed notebook, reproducibility manifest, and final model artefact should be supplied separately in the supplementary ZIP package. Individual-level NFHS-5/DHS microdata and record-level predictions are not included in this document.
